# Utilization of Wheat with Enhanced Carotenoid Levels and Various Fat Sources in Hen Diets

**DOI:** 10.3390/ani15091195

**Published:** 2025-04-23

**Authors:** Michaela Englmaierová, Jan Szmek, Miloš Skřivan, Pavel Horčička, Tomáš Taubner, Věra Skřivanová

**Affiliations:** 1 Department of Nutrition Physiology and Animal Product Quality, Institute of Animal Science, 104 00 Prague-Uhříněves, Czech Republic; szmek@af.czu.cz (J.S.); skrivan.milos@vuzv.cz (M.S.); taubner.tomas@vuzv.cz (T.T.); skrivanova.vera@vuzv.cz (V.S.); 2 Department of Microbiology, Nutrition and Dietetics, Faculty of Agrobiology, Food and Natural Resources, Czech University of Life Sciences Prague, 165 00 Prague, Czech Republic; 3 Selgen, a.s., Plant Breeding Station Stupice, 250 84 Sibřina, Czech Republic; horcicka@selgen.cz

**Keywords:** Pexeso wheat, fatty acids, tocopherols, lutein, zeaxanthin

## Abstract

In this study, we evaluated the effects of two wheat varieties with different carotenoid concentrations (Pexeso and Tercie) and two fat sources with contrasting fatty acid profiles (rapeseed oil and lard) on hen performance, egg quality, and egg yolk retention of carotenoids. The feed conversion ratio of hens that received Tercie wheat and lard in their diet were greater than those of hens that received other diets (*p* = 0.002). Greater (*p* ˂ 0.001) eggshell thickness and strength occurred when hens were fed a Pexeso wheat diet. Moreover, Pexeso wheat and lard increased lutein (*p* ˂ 0.001 and *p* = 0.001) and zeaxanthin (*p* ˂ 0.001 and *p* = 0.001) contents in egg yolks. The highest lutein retention (*p* = 0.010) occurred in the groups that received Pexeso wheat (46.4 and 47.4%), and the highest zeaxanthin retention (*p* = 0.011) occurred with a Pexeso wheat and lard diet (59.5%). The lowest lutein and zeaxanthin retention occurred in hens fed a Tercie wheat and rapeseed oil diet (23.6% for lutein retention and 24.1% for zeaxanthin retention). The Pexeso wheat and rapeseed oil diet increased the concentrations of α- and γ-tocopherol (*p* ˂ 0.001 and *p* ˂ 0.001) in egg yolks, which influenced the oxidative stability of the eggs. Compared with other diets, a Tercie wheat and rapeseed oil diet led to the lowest oxidative stability in fresh eggs (*p* = 0.041). In conclusion, Pexeso wheat had greater retention of biologically active substances and higher mineral contents than Tercie wheat, which was reflected in the performance of hens and the quality of eggs. The combination of Pexeso wheat with rapeseed oil, which is rich in tocopherols and polyunsaturated fatty acids and has a favorable n-6/n-3 ratio, increased the tocopherol content and the oxidative stability of egg yolk fats.

## Simple Summary

Most consumers evaluate the quality of eggs according to the color of the yolks. For this reason, carotenoids (mostly commercial pigments) are added to mixed feed for laying hens, of which wheat can be one of the components. Biofortification can be used to increase health-promoting substances in plants. Owing to this process, the carotenoid and mineral contents are higher in the new Pexeso variety of spring wheat. To meet the energy requirements of poultry, fats are also added to mixed feeds. In this study, we evaluated the effects of two wheat varieties with different carotenoid contents and two fat sources with different fatty acid profiles on hen performance and egg quality. Carotenoid retention in eggs and eggshell quality were greater when hens received Pexeso wheat. Lard also had a positive effect on the retention of carotenoids in the yolk. However, unlike rapeseed oil, lard has an inappropriate ratio of n-6/n-3 fatty acids and can contribute to the development of disease in humans. A combination of Pexeso wheat and rapeseed oil, which has high amounts of health-promoting polyunsaturated fatty acids and vitamin E, ensures the production of high-quality eggs.

## 1. Introduction

Carotenoids are a large group of pigments with important roles in humans and animals. They are divided into two subgroups according to the presence or absence of oxygen. Key carotenoids, including xanthophylls, lutein, and zeaxanthin, are found in plants and in some animal products. They are responsible for the color of the skin and the egg yolks of poultry. Grazing poultry obtain xanthophylls from pasture vegetation [1]. An increase in the content of xanthophylls in egg yolks has also been observed after the addition of alfalfa concentrate, tomato powder [2], yellow corn [3], colored carrot [4], or marigold flower [5] to the diet. On large farms without access to grazing or another natural source of xanthophylls, synthetic xanthophylls are added to poultry mixed feed. Xanthophylls are antioxidants, and xanthophyll supplementation in hen diets has been shown to increase the antioxidant capacity of the serum and liver and reduce malondialdehyde (MDA) production [6]. Lutein and zeaxanthin, which are ingested in mixed feeds, are emulsified with lipids and bile salts. They are then deposited in micelles, which are absorbed by diffusion in the duodenum and jejunum [7]. The source of lutein, the composition of the feed mixture, the content and type of fat, and the presence of phospholipids affect the stability and bioavailability of lutein and its storage in egg yolks [8,9,10].

Another option for increasing the carotenoid content in the yolk is to use a common feed component in which the carotenoid content has been increased by biofortification. Biofortification is a procedure that increases the concentration of essential nutrients in plants through agronomic intervention or genetic selection [11]. Wheat accounts for more than one-quarter of the dry matter production of field food crops and almost two-thirds of the daily human energy intake in several developing countries [12], and the nutritional value of wheat thus significantly affects human health [12,13]. The biofortification of wheat with mineral micronutrients has been successful and widespread. Using biofortification, Zou et al. [14] showed that increased concentrations of zinc (15-fold), iodine (13-fold), selenium (3.5-fold), and iron (by 12%) can occur. Examples of successful biofortification include golden rice, which produces β-carotene [15], or transgenic multivitamin corn, which provides β-carotene, zeaxanthin, lutein, lycopene, ascorbic acid, and folate [16]. In the present study, Pexeso wheat was tested, the lutein and zeaxanthin content of which was increased through a breeding process. The Pexeso wheat contained two and a half times more lutein and three and a half times more zeaxanthin compared to the normal carotenoid content of wheat, which is represented by the second wheat used, Tercie. Along with the carotenoid content, the mineral and tocopherol contents were also increased in the Pexeso wheat.

Fat is an important component of mixed feed. Fat is added to diets to increase energy and palatability and allows for better nutrient digestion and absorption [17]. Dietary fats are divided into vegetable fats and animal fats. The fat source modifies the fatty acid composition of the yolk [18]. From the perspective of animal and human health, a higher intake of polyunsaturated fatty acids, especially n-3 fatty acids, is desirable [19]. A rich source of n-3 fatty acids is primarily fish oil and flaxseed oil [20]. The content of n-3 fatty acids in egg yolk (linolenic acid, eicosapentaenoic acid, and docosahexaenoic acid) can also be increased by feeding hen diets enriched with rapeseed oil [21]. The disadvantage of polyunsaturated fatty acids is their higher susceptibility to oxidation than saturated fatty acids [22]. This is evident from the experiment of Gul et al. [23], where the addition of 6% rapeseed oil significantly increased thiobarbituric acid-reactive substance levels in eggs on the 21st day of the experiment. On the other hand, animal fat such as lard, which is rich in saturated fatty acids, is expected to have higher lipid oxidative stability. The addition of lard to the hen diet significantly improves yolk color [24].

Both the economy and efforts to reduce feed production costs play roles in the production of mixed feeds. Some vegetable oils and animal fats, which are usually less expensive but may adversely affect the fatty acid composition of animal products, are added to mixed feed to balance the metabolic energy requirements of poultry. As already mentioned, in terms of health, polyunsaturated fatty acids are especially valuable, but they are more susceptible to oxidation. However, carotenoids are effective antioxidants. Therefore, we assume that including a feed component rich in these antioxidants in hen diets will increase the content of bioactive substances in egg yolk and its oxidative stability. Therefore, the goal of this study was to determine the effects of two wheat varieties with different carotenoid concentrations and two fat sources with contrasting fatty acid profiles on hen performance, egg quality characteristics, and the retention of carotenoids in egg yolks.

## 2. Materials and Methods

### 2.1. Hens, Housing, Diets, and Performance

Two hundred and forty Lohmann Brown laying hens of 36 weeks of age were housed in enriched cages (ten hens per cage). The hens were assigned to 4 treatments with 6 replicates. The 2 × 2 factorial experiment included two wheat varieties (Pexeso or Tercie) and two fat sources (rapeseed oil or lard) in the mixed feed. A new Pexeso biofortified spring wheat variety with increased lutein and zeaxanthin concentrations was compared with the common Tercie wheat variety. Pexeso wheat was biofortified through a breeding process (variety code: 5095202; year of registration: 2018) and contained 1.115 mg/kg of lutein and 0.755 mg/kg of zeaxanthin, while the lutein and zeaxanthin contents in Tercie wheat were 0.439 and 0.214 mg/kg. Both wheat varieties were bred in the Czech Republic by the Selgen, a.s. company (Prague, Czech Republic). The diets and wheat variety compositions are shown in Table 1 and Table 2. The diets were provided in a mash form. Feed and fresh water were provided *ad libitum*. The technological and microclimatic conditions of housing corresponded to the management guide for the given hybrid. Room temperature was maintained at 20–22 °C with a relative humidity of 50% to 60%. The light cycle consisted of 14 h of light and 10 h of darkness. The light intensity ranged from 10 to 15 lux.

The experiment lasted 12 weeks (hen age: 36–48 weeks), including a preparatory period of 2 weeks. Data for determining performance characteristics were recorded and calculated weekly on a per-cage basis throughout the experiment except for the preparation period. The number of eggs per cage and feed intake per cage were recorded. The daily egg production was weighed three times a week; each egg was weighed separately. The daily egg mass output (g/hen/day) was calculated from the hen-day egg production and egg weight. The feed conversion ratio was calculated as kg feed/kg egg. The eggs used for chemical analyses of the yolk (vitamin and carotenoid contents and oxidative stability of fat) were collected at the end of the experiment from 48-week-old hens. Three yolks from each replicate were homogenized to form one sample (n = 6). Samples for the determination of carotenoid and vitamin contents were lyophilized and stored in a freezer at −24 °C until they were analyzed. The samples for the determination of the oxidative stability of the fat of the fresh eggs were taken and also stored in a freezer at −24 °C. To determine the oxidative stability of fat in the stored eggs, the eggs were first stored on paper trays for 28 days at a temperature of 18 °C and a relative humidity of 50–55%, and then, samples were taken and placed in a freezer (−24 °C).

### 2.2. Physical Analysis of Eggs

A whole day of egg production (from 48-week-old hens) was analyzed for the determination of physical parameters. The values were averaged per cage (n = 6). The eggs were weighed on a laboratory scale. Subsequently, the shell-breaking strength was evaluated on the vertical axis using an Instron 3360 apparatus (Instron, Norwood, MA, USA). The shell thickness was measured with a micrometer at the sharp and blunt ends and the equator after removing the shell membranes, and then, these values were averaged. The albumen and yolk percentages were determined by measuring the individual weight of each egg and the weights of these components. The yolk was weighed after the chalazae were removed. An IP54 digital micrometer head (Swiss Precision Instruments, Inc., Garden Grove, CA, USA) was used for the albumen height measurement. The Haugh units (HUs) were calculated from the egg weight and albumen height according to the following formula:

$$HU=100\times \log\;(H-1.7\times W^{0.37}+7.6)$$

where H = albumen height (mm), and W = egg weight (g).

The yolk color was evaluated visually with a DSM yolk color fan (DSM Nutritional Products, Basel, Switzerland).

### 2.3. Chemical Analyses of Diets and Eggs

Dry matter, fat, and crude protein contents were analyzed by standard AOAC [25] procedures. For the determination of phosphorus (P), calcium (Ca), and magnesium (Mg), dry homogenized samples were ashed at 550 °C, and the ash was dissolved in 3 M of hydrochloric acid. The total P in the solution was assayed using vanadate–molybdate reagent according to AOAC method No. 965.17 [25]. The Ca and Mg concentrations were determined by atomic absorption spectrometry using a Solaar M6 instrument (TJA Solutions, Cambridge, UK). The fatty acid (FA) composition was determined after chloroform–methanol extraction of total lipids [26]. Alkaline transmethylation of the FAs was then performed [27]. Gas chromatography of the methyl esters was performed using an HP 6890 chromatograph (Agilent Technologies, Inc., Santa Clara, CA, USA) with a programmed 60 m DB-23 capillary column and a flame ionization detector. The fatty acids were identified by their retention times compared with those of the standards.

The concentrations of lutein and zeaxanthin were analyzed with a high-performance liquid chromatography (HPLC) system (VP series; Shimadzu, Kyoto, Japan) according to a modified version of the method by Froescheis et al. [28]. The HPLC instrument was equipped with a diode array detector. A Kinetex C18 column (100 × 4.6 mm; 2.6 µm) supplied by Phenomenex (Torrance, CA, USA) was used. A gradient system was established, where eluent A was acetonitrile–water–ethyl acetate (88:10:2), and eluent B was acetonitrile–water–ethyl acetate (88:0:15).

Carotenoid, lutein, and zeaxanthin intakes were calculated based on the amount of carotenoids in the mixed feed and the average feed consumption per hen per day. Lutein and zeaxanthin were expressed in milligrams per kilogram of yolk dry matter. The retention of carotenoids in the yolk was calculated using the following formula:

R = (A × B × C)/D

where R = retention (%), A = egg mass production (g/hen/day), B = yolk percentage, C = carotenoid content in fresh yolk (mg/g), and D = carotenoid intake (mg/hen/day).

The α-tocopherol and γ-tocopherol contents were evaluated according to European standards EN 12822 [29] and EN 12823-1 [30], respectively, with an HPLC system (VP series; Shimadzu, Kyoto, Japan) equipped with a diode array detector.

The lipid peroxidation levels in the yolks of fresh and stored eggs were measured according to a modified version of the method by Czauderna et al. [31]. A Phenomenex C18 column (Synergi 2.5 µm, Hydro-RP, 100 Å, 100 mm × 3 mm; Phenomenex, Torrance, CA, USA) was used for chromatographic analysis (a Shimadzu HPLC system (VP series; Shimadzu, Kyoto, Japan) equipped with a diode-array detector). Solvent A consisted of water–acetonitrile (95:5, *v*/*v*), and solvent B consisted of acetonitrile. The lipid oxidative stability was expressed in mg of MDA per kg of eggs.

### 2.4. Statistical Analyses

The data were analyzed by using a two-way analysis of variance (ANOVA) with the general linear model (GLM) procedure in the SAS software 9.3 [32]. The main effects were wheat variety, fat source, and the interaction between these factors (wheat × fat). Differences between the groups were tested with Duncan’s multiple range test. The cage was the experimental unit (n = 6). The results are presented as the mean and the standard error of the mean (SEM), and a *p*-value < 0.05 was considered statistically significant.

## 3. Results

Pexeso wheat contained two and a half times more lutein and three and a half times more zeaxanthin than conventional Tercie wheat (Table 2). The new wheat variety also had higher fat, α-tocopherol, γ-tocopherol, and mineral contents.

Higher egg production (*p* = 0.039) was detected in hens that received a mixed feed with Pexeso wheat and lard than in hens that received Tercie wheat and lard or Pexeso wheat and rapeseed oil. The highest egg mass production (*p* < 0.001) and the heaviest eggs (*p* = 0.006) occurred in the treatment where hens received Tercie wheat and rapeseed oil (Table 3). Pexeso wheat resulted in reduced feed intake (*p* < 0.001). The highest feed conversion ratio (*p* = 0.002) was recorded in the treatment group that received Tercie wheat and lard in the diet. No mortality was recorded during the experiment.

The different diets had no effect on Haugh units, albumen percentage, or yolk percentage (Table 4). Pexeso wheat enhanced the yolk color (*p* ˂ 0.001), the yolk percentage (*p* ˂ 0.001), the thickness of the eggshell (*p* ˂ 0.001), and the strength of the eggshell (*p* ˂ 0.001).

The values for carotenoid intake (Table 5) were consistent with their concentrations in the diet; carotenoid concentrations were approximately double in the diets that contained Pexeso wheat (Table 2). This was also reflected by individual carotenoid contents (*p* ˂ 0.001) in the egg yolks. The inclusion of rapeseed oil in the mixed feed reduced (*p* = 0.001) lutein and zeaxanthin contents in the yolks. The greatest lutein retention (*p* = 0.010) was recorded in the treatments that received Pexeso wheat (46.4 and 47.4%), and the lowest lutein retention occurred in hens that received Tercia wheat and rapeseed oil (23.6%). The greatest zeaxanthin retention (*p* = 0.011) occurred in the treatment that received Pexeso wheat and lard (59.5%), and the lowest zeaxanthin retention occurred in hens that received Tercia wheat and rapeseed oil (24.1%).

Hens that received mixed feed with rapeseed oil and Pexeso wheat produced eggs with increased concentrations of α- and γ-tocopherol (*p* ˂ 0.001 and *p* = 0.002, respectively) in the egg yolks (Table 6). The oxidative stability of fresh egg yolks was the lowest when hens were fed the diet with Tercie wheat and rapeseed oil (*p* = 0.041). The formation of MDA oxidation products in stored eggs decreased when hens were fed the mixed feed diet with Pexeso wheat (*p* = 0.006) and lard (*p* = 0.004).

## 4. Discussion

Wheat–soybean mixed feed is common in hen farming because it ensures high performance. Recently, wheat cultivars have been bred to have a blue to red aleurone layer or pericarp, which is due to anthocyanins. In contrast, carotenoids color the endosperm yellow [33]. Wheat is a cereal grain with a low concentration of fat, and the fat concentration is associated with the concentration of xanthophylls, which are lipophilic substances. In biofortified Pexeso wheat, lutein has been increased two and a half-fold, and zeaxanthin has been increased three and a half-fold, compared to Tercie wheat. Marigold flowers are a common organic source of these dietary carotenoids for laying hens. The concentrations of lutein and zeaxanthin in commercial marigold-based products are many times greater than those in biofortified Pexeso wheat [34]. In contrast, carotenoid deposition in the yolk, based on the amount of carotenoids in a diet supplemented with marigold or Pexeso wheat, is greater for Pexeso wheat diets. Through the biofortification of a common maize variety, Naqvi et al. [35] showed that the lutein concentration increased by 30%, from 8.74 mg/g to 11.36 mg/g, and the zeaxanthin concentration increased fourfold, from 2.05 mg/kg to 8.19 mg/kg. The initial concentration of lutein in maize was 19.9 times greater than that in wheat, and that of zeaxanthin was 9.6 times greater than that of wheat. Unlike wheat, maize has a relatively high fat content. Using Pexeso wheat in wheat–soybean mixed feeds, which are common in Europe, will not ensure optimal egg yolk coloration according to the requirements of many consumers, and it will be necessary in this case to add a source of color pigments, such as marigold flowers. Pexeso wheat has experienced good sales in the Czech Republic and abroad in the food industry. According to our observations (unpublished data), the increase in yellowness in cake from Pexeso wheat reaches 11%.

The lower feed intake and conversion that were observed in hens fed Pexeso wheat was in accordance with the findings of Nabi et al. [36], who reported that dietary supplementation with carotenoids improved the production and health of poultry. In addition, carotenoids have an anti-inflammatory effect [37] and can regulate the immune response [38], which can also affect performance. However, in the present study, there was no influence on mortality as an indicator of poultry health. A positive effect of carotenoids on performance was also demonstrated by Saleh et al. [39], who tested a natural colorant, paprika, in a hen diet. Likewise, Cui et al. [40] investigated the effects of the dietary inclusion of alfalfa meal on laying performance in hens. A meta-analysis by Yunitasari et al. [41] suggests that carotenoid supplementation can improve productivity, increase egg quality, and improve immunity.

Including Pexeso wheat in the diet of hens increased the eggshell thickness and strength. Eggshell strength (an important characteristic of eggs) and eggshell thickness in the treatments that received Pexeso wheat may have been related to higher Mg concentrations, which is consistent with the results of Kim et al. [42], Belkameh et al. [43], and Kim et al. [44]. The greater deposition of lutein and zeaxanthin in egg yolks was due to a higher dietary concentration of both xanthophylls in Pexeso wheat, and this observation was supported by greater retention in the groups that were fed this wheat variety. Lipids have a positive effect on the absorption of lipophilic carotenoids. Compared with fats rich in monounsaturated and polyunsaturated fatty acids, dietary fats rich in saturated fatty acids have been shown to increase the availability of lutein and zeaxanthin [45], which is consistent with our findings in this study. Similarly, Conlon et al. [46] reported that coconut oil enhanced the tissue uptake of tomato carotenoids to a greater degree than safflower oil, and Hu et al. [47] tested the intestinal absorption of β-carotene ingested with a meal rich in beef tallow or sunflower oil. In contrast, Marounek et al. [48] found that the effect of dietary fat saturation on the deposition of carotenoids in yolks was nonsignificant or small.

Compared with lard, rapeseed oil has a greater amount of vitamin E, which was reflected by the greater amount of this vitamin in the yolks of eggs from hens that were fed a diet with rapeseed oil. Moreover, supplementation with a fat rich in unsaturated fatty acids leads to greater vitamin E bioavailability than supplementation with a fat rich in saturated fatty acids [49]. In addition, Pexeso wheat, which is rich in carotenoids, had a positive effect on the γ-tocopherol content in eggs compared to the effect of Tercie wheat. This finding may be related to the antioxidant properties of carotenoids, which protect γ-tocopherol from oxidative degradation in the upper gastrointestinal tract [50]. In contrast, carotenoids may compete with γ-tocopherol for incorporation into micelles or uptake by putative transporters, thus impairing absorption [51]. Woodall et al. [52] did not observe an effect of carotenoids on tissue α-tocopherol levels.

The increased retention of lutein and zeaxanthin likely increased the oxidative stability of egg yolks because these natural lipophilic pigments are powerful antioxidants. The increased oxidative stability of egg yolks due to lutein and zeaxanthin has been demonstrated by Untea et al. [53], Kljak et al. [5], Liu et al. [54], and Kusumiyati et al. [34]. Additionally, the treatment groups that received lard presented lower MDA production than the groups that received rapeseed oil, and this was related to a greater proportion of saturated fatty acids, which are less susceptible to oxidation than the polyunsaturated fatty acids in rapeseed oil.

## 5. Conclusions

Increasing the carotenoid content in mixed feed is desirable in the context of animal health and in the production of quality products that benefit human health. Including Pexeso wheat, which has higher carotenoid content than Tercie wheat, in the diets of hens improved the feed conversion ratio and the quality of eggs due to the greater retention of biologically active substances, particularly carotenoids. In terms of yolk color, the addition of Pexeso wheat to mixed feed was not sufficient to meet consumer requirements. A greater retention of carotenoids occurred when lard was included as a fat source in the hen diet; however, the addition of rapeseed oil resulted in a favorable ratio of n-6 to n-3 fatty acids in the diet (approximately four) and an increase in vitamin E—which has antioxidant effects—in the yolks. The combination of rapeseed oil and Pexeso wheat ensures sufficient protection of the fat in the yolk from oxidation. In addition, Pexeso wheat stands out for its high Mg content, which has a positive effect on shell quality and can affect the economics of production by reducing the incidence of broken eggs. Therefore, we recommend using Pexeso wheat, in combination with rapeseed oil as a source of fat, in a mixed feed diet for hens. Further research should be focused on breeding wheat varieties with higher carotenoid content to ensure optimal yolk coloration in terms of consumer requirements.

## Figures and Tables

**Table 1:** Ingredients (g/kg) and nutrient compositions of the diets.

Wheat Variety	Tercie		Pexeso	
Fat Source	Rapeseed Oil	Lard	Rapeseed Oil	Lard
Tercie wheat, g/kg	652.2	652.2	-	-
Pexeso wheat, g/kg	-	-	631.5	631.5
Soybean meal, g/kg	176.0	176.0	189.0	189.0
Limestone, 1–2 mm, g/kg	96.0	96.0	96.0	96.0
Rapeseed oil, g/kg	54.0	-	62.0	-
Lard, g/kg	-	54.0	-	62.0
Monocalcium phosphate, g/kg	10.5	10.5	10.5	10.5
Vitamin–mineral premix ^1^, g/kg	5.0	5.0	5.0	5.0
Sodium bicarbonate, g/kg	2.6	2.6	2.6	2.6
Sodium chloride, g/kg	1.9	1.9	1.9	1.9
DL-Methionine, g/kg	1.1	1.1	1.1	1.1
L-Lysine, g/kg	0.5	0.5	0.3	0.3
L-Threonine, g/kg	0.2	0.2	0.1	0.1
Analyzed nutrient content (g/kg)				
Crude protein, g/kg	162.2	162.4	161.9	162.0
Ether extract, g/kg	34.3	34.5	38.4	38.5
ME_N_, MJ/kg	11.48	11.52	11.47	11.51
α-Tocopherol, mg/kg	34.34	26.41	36.33	25.10
γ-Tocopherol, mg/kg	13.16	3.91	14.19	3.73
Lutein, mg/kg	0.439	0.379	0.745	0.806
Zeaxanthin, mg/kg	0.240	0.229	0.468	0.452
SFA, mg/100 g	649	1826	639	1725
MUFA, mg/100 g	2153	1726	2176	1584
PUFA, mg/100 g	2125	1411	2113	1340
PUFA n-6/n-3 ratio	3.9	7.1	4.4	7.5
Calcium, g/kg	39.0	38.9	39.2	39.1
Available phosphorus, g/kg	3.91	3.88	4.03	4.01
Magnesium, g/kg	2.26	2.28	3.12	3.15

^1^ Vitamin–mineral premix provided per kg of mixed diet: vitamin A—8721 IU, vitamin D3—3000 IU, niacin—25 mg, calcium pantothenate—8 mg, thiamine—2 mg, riboflavin—5 mg, pyridoxin—4 mg, folic acid—0.5 mg, biotin—0.075 mg, cobalamin—0.01 mg, choline chloride—250 mg, menadione—2 mg, betaine—100 mg, butylated hydroxytoluene—7.5 mg, ethoxyquin—5.6 mg, butylhydroxyanisole—1 mg, DL-methionine—0.7 g, manganese—70 mg, zinc—50 mg, iron—40 mg, copper—6 mg, iodine—1 mg, cobalt—0.3 mg, selenium—0.2 mg. ME_N_: metabolizable energy; SFA: saturated fatty acid; MUFA: monounsaturated fatty acid; PUFA: polyunsaturated fatty acid.

**Table 2:** The nutrient compositions of the wheat varieties.

Wheat Variety	Tercie	Pexeso
Crude protein, g/kg	129.0	123.9
Ether extract, g/kg	14.3	15.7
ME_N_, MJ/kg	12.07	11.82
α-Tocopherol, mg/kg	5.03	5.44
γ-Tocopherol, mg/kg	2.38	2.65
Lutein, mg/kg	0.439	1.115
Zeaxanthin, mg/kg	0.214	0.755
SFA, mg/100 g	289	360
MUFA, mg/100 g	235	344
PUFA, mg/100 g	826	866
PUFA n-6/n-3 ratio	7.3	11.1
Calcium, g/kg	0.66	0.84
Available phosphorus, g/kg	2.18	2.41
Magnesium, g/kg	2.4	3.8

ME_N_: metabolizable energy; SFA: saturated fatty acid; MUFA: monounsaturated fatty acid; PUFA: polyunsaturated fatty acid.

**Table 3:** Effects of two wheat varieties and two fat sources on the performance of hens (n = 6).

		Hen-Day Egg Production, %	Egg Mass, g/hen/day	Egg Weight, g	Feed Intake, g/hen/day	Feed Conversion Ratio, kg/kg
Wheat variety	Tercie	93.9	59.5	63.3 ^a^	129.0 ^a^	2.17 ^a^
	Pexeso	94.5	59.0	62.5 ^b^	123.5 ^b^	2.09 ^b^
Fat source	Rapeseed oil	94.0	60.0 ^a^	63.9 ^a^	126.4	2.11 ^b^
	Lard	94.5	58.5 ^b^	61.9 ^b^	126.0	2.16 ^a^
Wheat variety	Fat source					
Tercie	Rapeseed oil	94.2 ^ab^	60.9 ^a^	64.7 ^a^	128.8	2.12 ^b^
	Lard	93.7 ^b^	58.1 ^c^	62.0 ^c^	129.1	2.23 ^a^
Pexeso	Rapeseed oil	93.7 ^b^	59.1 ^b^	63.0 ^b^	124.1	2.10 ^b^
	Lard	95.2 ^a^	58.9 ^bc^	61.9 ^c^	122.8	2.09 ^b^
SEM		0.25	0.20	0.17	0.50	0.011
*p*-Value	Wheat	NS	NS	0.008	<0.001	<0.001
	Fat	NS	<0.001	<0.001	NS	0.010
	Wheat × fat	0.039	<0.001	0.006	NS	0.002

^a–c^ Means in the same row with different superscripts differ significantly; SEM: standard error of the mean; NS: not significant.

**Table 4:** Effects of two wheat varieties and two fat sources on the physical characteristics of egg quality (n = 6).

		Haugh Units	Albumen Percentage, %	Yolk Percentage, %	DSM Yolk Color Fan	Shell Percentage, %	Shell Thickness, µm	Shell Breaking Strength, N
Wheat variety	Tercie	86.7	64.1	25.8	1.55 ^b^	10.1 ^b^	353 ^b^	46.5 ^b^
	Pexeso	85.5	63.7	25.9	3.40 ^a^	10.5 ^a^	365 ^a^	49.8 ^a^
Fat source	Rapeseed oil	85.9	64.0	25.7	2.41	10.3	361	48.5
	Lard	86.3	63.7	26.0	2.53	10.3	356	47.8
Wheat variety	Fat source							
Tercie	Rapeseed oil	86.3	64.5	25.5	1.48	10.0	356	47.1
	Lard	87.1	63.7	26.2	1.62	10.1	349	45.9
Pexeso	Rapeseed oil	85.6	63.6	25.9	3.35	10.5	367	49.9
	Lard	85.5	63.7	25.9	3.45	10.4	364	49.7
SEM		0.39	0.13	0.11	0.072	0.05	1.97	0.45
*p*-Value	Wheat	NS	NS	NS	<0.001	<0.001	<0.001	<0.001
	Fat	NS	NS	NS	NS	NS	NS	NS
	Wheat×fat	NS	NS	NS	NS	NS	NS	NS

^a,b^ Means in the same row with different superscripts differ significantly; SEM: standard error of the mean; NS: not significant.

**Table 5:** Carotenoid intake, content, and retention in egg yolks (n = 6).

		Lutein Intake, mg/hen/day	Lutein Content in Yolk, mg/kg DM	Lutein Retention in Yolk, %	Zeaxanthin Intake, mg/hen/day	Zeaxanthin Content in Yolk, mg/kg DM	Zeaxanthin Retention in Yolk, %
Wheat variety	Tercie	0.053	1.80 ^b^	27.0 ^b^	0.030	1.24 ^b^	32.1 ^b^
	Pexeso	0.096	5.77 ^a^	46.9 ^a^	0.057	4.11 ^a^	56.5 ^a^
Fat source	Rapeseed oil	0.074	3.59 ^b^	35.0 ^b^	0.044	2.46 ^b^	38.8 ^b^
	Lard	0.074	3.98 ^a^	38.9 ^a^	0.043	2.89 ^a^	49.9 ^a^
Wheat variety	Fat source						
Tercie	Rapeseed oil	0.057	1.69	23.6 ^c^	0.031	0.94	24.1 ^d^
	Lard	0.049	1.91	30.4 ^b^	0.030	1.53	40.2 ^c^
Pexeso	Rapeseed oil	0.092	5.49	46.4 ^a^	0.058	3.98	53.5 ^b^
	Lard	0.099	6.04	47.4 ^a^	0.056	4.25	59.5 ^a^
SEM		-	0.418	2.19	-	0.309	2.96
*p*-Value	Wheat	-	<0.001	<0.001	-	<0.001	<0.001
	Fat	-	0.001	0.001	-	0.001	<0.001
	Wheat×fat	-	NS	0.010	-	NS	0.011

^a–d^ Means in the same row with different superscripts differ significantly; SEM: standard error of the mean; NS: not significant; DM: dry matter.

**Table 6:** Vitamin and MDA concentrations in egg yolks (n = 6).

		α-Tocopherol, mg/kg DM	γ-Tocopherol, mg/kg DM	MDA Day 0, mg/kg	MDA Day 28, mg/kg
Wheat variety	Tercie	146.2	13.84 ^b^	0.412 ^a^	0.434 ^a^
	Pexeso	157.7	15.54 ^a^	0.342 ^b^	0.390 ^b^
Fat source	Rapeseed oil	165.2 ^a^	20.30 ^a^	0.398 ^a^	0.436 ^a^
	Lard	138.6 ^b^	9.09 ^b^	0.356 ^b^	0.389 ^b^
Wheat variety	Fat source				
Tercie	Rapeseed oil	156.3 ^b^	17.87 ^b^	0.448 ^a^	0.458
	Lard	136.0 ^d^	9.81 ^c^	0.375 ^b^	0.410
Pexeso	Rapeseed oil	174.1 ^a^	22.72 ^a^	0.347 ^b^	0.413
	Lard	141.2 ^c^	8.36 ^d^	0.337 ^b^	0.368
SEM		3.35	1.247	0.0112	0.0095
*p*-Value	Wheat	NS	<0.001	<0.001	0.006
	Fat	<0.001	<0.001	0.008	0.004
	Wheat×fat	<0.001	0.002	0.041	NS

^a–d^ Means in the same row with different superscripts differ significantly; SEM: standard error of the mean; NS: not significant; DM: dry matter; MDA: malondialdehyde.

## Data Availability

The data presented in this study are available upon request from the corresponding author.
